# The Effects of Acute and Chronic Alcohol Administration and Withdrawal on Bone Microstructure, Mechanical Strength, and Remodeling Protein Expression and Their Relation to an Antioxidant and FGF23 In Vivo

**DOI:** 10.3390/biomedicines12071515

**Published:** 2024-07-08

**Authors:** Syed Alhafiz Syed Hashim, Isa Naina Mohamed, Norazlina Mohamed

**Affiliations:** 1 Department of Pharmacology, Faculty of Medicine, Universiti Kebangsaan Malaysia, Cheras, Kuala Lumpur 56000, Malaysia; alhafizsyed@gmail.com (S.A.S.H.); isanaina@ppukm.ukm.edu.my (I.N.M.); 2 Institute of Pharmaceutical Science, King’s College London, Franklin-Wilkins Building, 150 Stamford Street, London SE1 9NH, UK

**Keywords:** ethanol, osteoporosis, osteopenia, bone micro-CT, bone remodeling

## Abstract

Alcohol’s detrimental effects on bone health are well established, yet some literature suggests moderate consumption may offer benefits. With alcohol use on the rise, we investigate the impact of acute and chronic alcohol administration, along with withdrawal, on male Wistar rat femurs. We observed a transient cortical thickness increase with acute alcohol (AA) compared to chronic exposure (CA) but no significant changes in trabecular parameters or mechanical properties. High osteocalcin and osteopontin expression levels were noted in AA, alongside elevated RANKL expression. Conversely, CA showed low TRAP levels. FGF23 expression significantly increased during alcohol withdrawal (AW), while GPX decreased after chronic exposure but rose during withdrawal. Although mechanical strength changes were insignificant, biochemical shifts suggest alcohol exposure promotes bone resorption, reduces antioxidant protection, and potentially hampers active vitamin D and phosphate reabsorption via FGF23 upregulation.

## 1. Introduction

Alcohol consumption presents a pressing global health challenge, contributing to three million deaths and more than 5% of the global disease burden in 2016 [1]. Alarmingly, 75% of these fatalities occur among men, with approximately 43 million individuals worldwide affected by alcohol use disorder (AUD) [2]. Although alcohol consumption has always been associated with cardiovascular [3,4] and liver diseases [5], it also poses significant risks to bone health [6,7]. Bone is a living and growing tissue that constantly undergoes a remodeling process throughout life through interaction between cells, hormones, and vitamins [8]. The remodeling process predominantly involves osteoblasts, the cells responsible for forming new bone in osteoblastogenesis, and osteoclasts, the cells that break down bone in osteoclastogenesis [9]. Both bone formation and resorption need to occur in balance to maintain optimal bone structure. An imbalance in the bone remodeling process, whereby bone resorption exceeds bone formation, can lead to osteoporosis [10]. Osteoporosis is characterized by low bone mineral density and altered bone microarchitecture, resulting in bone fragility and an increased risk of fractures. Bone microstructure and strength are crucial for maintaining bone health and significantly influence its ability to resist fractures and maintain overall structural integrity. While dual-energy X-ray absorptiometry (DXA) remains the gold standard for diagnosing osteoporosis [11], it may not detect changes in bone microstructure. Micro-CT offers superior resolution for precise morphometric analyses [12] and provides deeper insights into structural alterations, and therefore, it is utilized in this study. Numerous preventive strategies have been outlined for osteoporosis, one of which includes a reduction in alcohol consumption [13]. Alcohol is known to have detrimental effects on bone by exerting direct toxic effects on bone cells [14,15,16] and indirectly disrupting bone remodeling balance by affecting various hormones such as growth and estrogen hormones [17]. It interrupts calcium and bone homeostasis, leading to disturbances in bone growth [18]. Alcohol affects bone metabolism by increasing parathyroid hormone release, which stimulates osteoclast activity [19] and bone resorption. It also reduces the number of osteoblasts [14,20], thereby decreasing bone formation. These dual effects lead to decreased bone density and an increased risk of osteoporosis over time. Additionally, alcohol has been reported to decrease the levels of activated vitamin D, resulting in low calcium absorption by the intestine and increased bone resorption to restore serum calcium levels [21]. Furthermore, alcohol metabolism produces reactive oxygen species (ROS) and reactive nitrogen species (RNS) while depleting antioxidant levels, all of which contribute to detrimental effects on bone [22]. Various bone markers are used to determine bone remodeling activity. Bone formation markers include osteocalcin (OCN), bone alkaline phosphatase (ALP), procollagen type 1 carboxy-terminal propeptide (P1CP), and procollagen type 1 N-propeptide (PINP) [23], whereas markers of bone resorption include receptor activator of nuclear factor kappa-B ligand (RANKL), tartrate-resistant acid phosphatase (TRAP), and telopeptides of type 1 collagen (CTX-1) [24].

Fibroblast growth factor 23 (FGF23) is a hormone encoded by the FGF23 gene located on chromosome 12p13 [25]. It is primarily synthesized in the bone by osteocytes [26] and plays an important role in regulating phosphate and vitamin D metabolism [27]. FGF23 acts as a phosphaturic factor, leading to increased renal phosphate excretion and the suppression of 1,25(OH)2-vitamin D3 [27]. This hormone inhibits bone formation and mineralization, significantly influencing bone health [28]. Additionally, FGF23 directly inhibits the differentiation of osteoprogenitor cells, which affects bone development [29]. Alcohol has been reported to induce upregulation of hepatic FGF23 and plasma FGF23 levels in patients with cirrhosis [30]. Chronic alcohol consumption is also associated with FGF23-related hypophosphatemic osteomalacia [31]. Interestingly, alcohol abstinence has been shown to decrease FGF23 levels after five months [31]. However, more research is needed to fully understand the mechanisms and implications of these interactions.

Despite numerous studies delineating the harmful effects of alcohol on bone [17,32], some research suggests that moderate alcohol consumption could potentially benefit bone health [33,34]. Since there is still a paucity of information regarding the relationship between alcohol and bone, this study was intended to investigate the effects of acute and chronic alcohol administration and withdrawal on bone microstructure, strength, and bone remodeling protein expression, as well as the relationship between these effects and the FGF23 protein and an antioxidant. In this study, we utilized an ethanol treatment protocol in rats as described in previous literature [35]. This model was used to mimic the chronic and relapsing nature of human alcohol consumption patterns, providing a more comprehensive understanding of the long-term effects of alcohol on bone health. This approach allowed us to study both the acute and chronic effects of alcohol consumption and withdrawal on bone tissue.

## 2. Materials and Methods

### 2.1. Instruments and Kits

An Omni Bead Ruptor 24 (Omni International Inc., Kennesaw, GA, USA) was used to homogenize the samples. An RTX high-performance rotary tool (Black & Decker, New Britain, CT, USA) was utilized to cut the femurs. Samples were centrifuged using a Microfuge 22R centrifuge (Beckman Coulter Inc., Brea, CA, USA). Imaging was carried out using a micro-computed tomography (micro-CT) Skyscan 1076 scanner (Skyscan, Kartuizersweg Kontich, Belgium). Mechanical strength testing of the samples was performed using a universal mechanical strength testing machine (Autograph AGS-X 500N, Shimadzu, Kyoto, Japan). For biochemical analyses, a multimode plate reader (Thermo Fisher Scientific, Waltham, MA, USA) was employed. ELISA kits from Elabscience, Houston, TX, USA were used, including the Rat Osteopontin ELISA Kit (OPN) (E-EL-R0702), the Rat Osteocalcin ELISA Kit (OC/BGP) (E-EL-R0243), the Rat RANKL ELISA Kit (E-EL-R0841), the Rat Fibroblast Growth Factor 23 (FGF23) ELISA Kit (E-EL-R2410), the Rat Tartrate Resistant Acid Phosphatase (TRAP) ACP5 ELISA Kit (E-EL-R0939), and the Rat Glutathione Peroxidase (GPx) ELISA Kit (E-EL-R2491).

### 2.2. Animals and Treatment

This study examined 24 femur bone specimens from male Wistar rats aged two months old, weighing around 300 g, retrieved from the Laboratory Animal Resource Unit of Universiti Kebangsaan Malaysia (LARU). The rats were randomly divided into four groups with *n* = 6 in each group: normal control (NC), acute alcohol (AA), chronic alcohol (CA), and alcohol withdrawal (AW). They were individually housed under a 12 h light–dark cycle at a constant temperature of 24 °C and acclimatized for one week upon arrival.

The normal control and acute alcohol groups received a modified liquid diet (MLD) without ethanol for 27 days and intraperitoneal normal saline and ethanol (2.5 g/kg, 20% *V*/*V*) on day 28, 60 min before humane culling. Chronic alcohol administration was given following Kumar et al.’s technique [35]. The chronic alcohol group received MLD without ethanol for the first 7 days. From day 8, ethanol was gradually introduced: 2.4% for 3 days, 4.8% on day 11 for 3 days, and 7.2% until day 27. On day 28, the members of this group received intraperitoneal ethanol (2.5 g/kg, 20% *V*/*V*) 60 min before culling. The withdrawal group received the same treatment as the chronic alcohol group but was culled 6 h after intraperitoneal ethanol (2.5 g/kg, 20% *V*/*V*) administration. The left femurs were collected, cleaned, and wrapped in gauze soaked in phosphate buffer solution (PBS) and aluminum foil before storage at −70 °C. The experimental protocol was approved by the Universiti Kebangsaan Malaysia Animal Ethics Committee (UKMAEC) (approval code: FAR/PP/2019/NORAZLINA/30-OCT./1050-OCT-2019-MAR-2020-AR-CAT2).

### 2.3. Micro-Computed Tomography (µCT) Analysis of Femur

The scanning and analysis of harvested left femurs were performed using a µCT Skyscan 1076 Scanner and CTAn software (Skyscan, Kartuizersweg Kontich, Belgium). The selected µCT parameters for scanning were as follows: X-ray voltage = 92 kV, X-ray current = 100 µA, image pixel size = 9 µm, rotation step = 0.5° with high camera resolution. After scanning, 200 slices of the volume of interest (VOI) were selected for both trabecular and cortical bone, with reference to the distal growth plate for analysis. The measurement of both trabecular and cortical areas commenced at approximately 1.0 mm and 7.0 mm, respectively, from the distal growth plate, extending towards the proximal end of the femur. Parameters measured in trabecular bone included trabecular thickness (Tb.Th, unit = mm), trabecular separation (Tb.Sp, unit = mm), trabecular number (Tb.N, unit = 1/mm), connectivity density (Conn. D, unit = 1/mm^3^), structural model index (SMI), and trabecular bone volume (BV/TV, unit = %). Parameters measured in cortical bone included cortical thickness (Ct.Th, unit = mm), total cross-sectional area (Tt.Ar, unit = mm^2^), cortical bone area (Ct.Ar, unit = mm^2^), and cortical area fraction (Ct Ar/Tt.Ar, unit = %)

### 2.4. Bone Biomechanical Strength Analysis of Femur

The biomechanical strength of the left femurs was evaluated through a three-point bending test using the Shimadzu Universal Testing Machine (Autograph AGS-X 500N, Kyoto, Japan). The speed was set at 5 mm/min with a span length of 10 mm apart. The left femurs were mounted on two supporting rods in a position where the anterior surface of the bone faced upward. The load was applied directly to the midpoint of the anterior surface of the femur until it broke. The results were analyzed using Trapezium Lite X software (https://www.shimadzu.com/an/products/materials-testing/uni-ttm-software/trapezium-lite-x/index.html, accessed on 26 June 2024). The parameters measured were load (unit = N), displacement (unit = mm), stress (unit = N/mm^2^), strain (unit = %), stiffness (unit = N/mm), and Young’s modulus of elasticity (unit = N/mm^2^).

### 2.5. Enzyme-Linked Immunosorbent Assay (ELISA)

The left femur samples were thawed at room temperature and cut into 100 mg pieces using an RTX high-performance rotary tool (Black & Decker, New Britain, CT, USA). The samples were homogenized in 4 mL PBS using an Omni Ruptor (Omni International Inc., Kennesaw, GA, USA) and subsequently centrifuged at 1600 rpm and 4 °C for 10 min using a Microfuge 22R centrifuge (Beckman Coulter Inc., Brea, California, USA). The clear supernatant of each homogenate was transferred to ELISA wells. Osteocalcin (OCN) protein expression was measured using the Rat Osteocalcin (OC/BGP) ELISA kit (Elabscience, E-EL-R0243), while receptor activator of nuclear factor kappa-Β ligand (RANKL) protein expression was measured using the Rat RANKL ELISA kit (Elabscience, E-EL-R0841). Rat fibroblast growth factor 23 (FGF23) protein expression was measured using the Rat FGF23 ELISA Kit (Elabscience, E-EL-R2410), Rat tartrate-resistant acid phosphatase (TRAP) ACP5 protein expression was measured using the Rat TRAP ACP5 ELISA Kit (Elabscience, E-EL-R0939), and rat glutathione peroxidase (GPx) protein expression was measured using the Rat GPx ELISA Kit (Elabscience, E-EL-R2491). All procedures were performed as per the respective protocols.

### 2.6. Statistical Analysis

The data were analyzed using the Statistical Package for the Social Sciences (SPSS) version 26 software (IBM, Armonk, NY, USA) and GraphPad Prism 10. Data distribution was determined by the Shapiro–Wilk Test of Normality. The statistical tests used in this study were One-Way Analysis of Variance (ANOVA) with Tukey post hoc test for normally distributed data and the Kruskal–Wallis test with pairwise comparison for non-parametric data. All data are presented as the mean and standard error of the mean (SEM). A *p*-value of less than 0.05 was considered statistically significant.

## 3. Results

### 3.1. Trabecular Bone Parameters

There were no significant differences observed in all trabecular bone parameters among the groups as depicted in Table 1. The acute alcohol (AA) group showed slightly higher connectivity density [703.71 (75.17) 1/mm^3^] compared to the normal control (NC) [632.8 (63.01) 1/mm^3^], chronic alcohol (CA) [678.23 (77.24) 1/mm^3^], and alcohol withdrawal (AW) [690.34 (95.94) 1/mm^3^], whereas the AA group demonstrated the lowest bone volume to total volume ratio (BV/TV) [5.51(0.69) %] and trabecular thickness (Tb.Th) [0.039 (0.001) mm] across all experimental and control groups.

### 3.2. Cortical Bone Parameters

**Figure 1:**
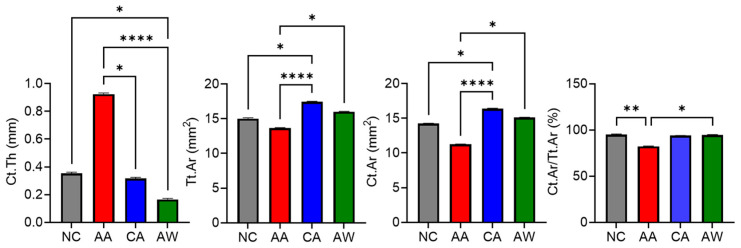
The effects of alcohol administration and withdrawal on the cortical bone of the left femur. Abbreviations: NC: normal control group; AA: acute alcohol group; CA: chronic alcohol group; AW: alcohol withdrawal group. Ct.Th: cortical thickness, Tt.Ar: total cross-sectional area, Ct.Ar: cortical area, Ct.Ar/Tt.Ar: cortical area to total area ratio. The results are presented as mean and standard error of mean for 6 replicates. * *p* < 0.05, ** *p* < 0.01, and **** *p* < 0.0001 indicate significant differences. The statistical test was performed using GraphPad Prism 10.

For cortical bone microstructure, the AA group exhibited a significantly higher (*p* < 0.05) Ct.Th [0.93 (0.03)] compared to the other groups: NC [0.36 (0.01)], CA [0.31 (0.04)], and AW [0.16 (0.05)]. Additionally, the AA group showed significantly (*p* < 0.0001) lower total cross-sectional area (Tt.Ar) [13.53 (0.55)] and cortical area (Ct.Ar) [11.13 (0.29)] (*p* < 0.0001) compared to the CA group, with Tt.Ar and Ct.Ar values of [17.44 (0.65)] and [16.37 (0.54)], respectively. Furthermore, the cortical area fraction (Ct.Ar/Tt.Ar) was significantly lower in the AA group [82.93 (4.12)] (*p* < 0.01) compared to the NC, CA, and AW groups, with [94.92 (0.94)], [93.94 (0.58)], and [93.78 (0.56)], respectively, as shown in Figure 1.

### 3.3. Bone Biomechanical Strength

Although no statistical significance was found in any group for all parameters, both AA and CA groups consistently exhibited higher load, stress, displacement, strain, stiffness, elasticity, and Young’s modulus compared to the NC group. Conversely, AW demonstrated lower values for all parameters compared to CA, as shown in Table 2.

### 3.4. Protein Expression of Bone Remodeling Markers, FGF23, and Antioxidant

**Figure 2:**
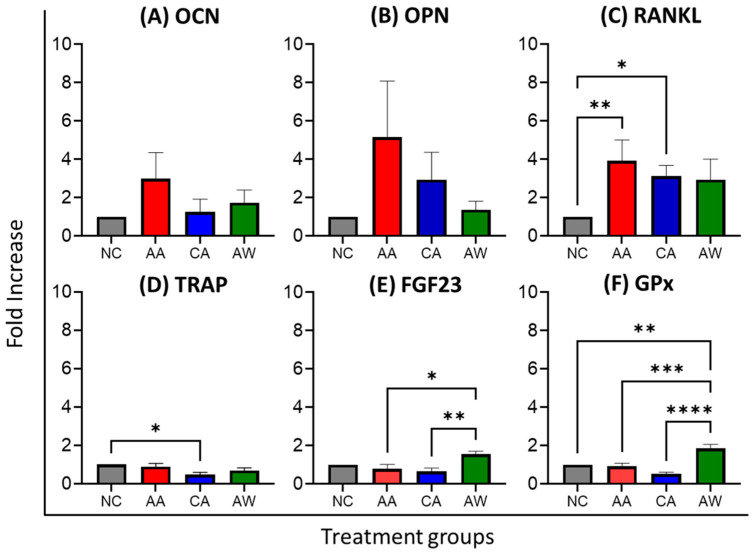
The effects of alcohol administration and withdrawal on bone remodeling markers, an antioxidant, and FGF23. Abbreviations: NC: normal control group; AA: acute alcohol group; CA: chronic alcohol group; AW: alcohol withdrawal group. OCN: osteocalcin, OPN: osteopontin, RANKL: receptor activator of nuclear factor kappa-Β ligand, TRAP: tartrate-resistant acid phosphatase, FGF23: fibroblast growth factor 23, GPx: glutathione peroxidase. * *p* < 0.05, ** *p* < 0.01, *** *p* < 0.001, and **** *p* < 0.0001 indicate significant differences. The statistical test was performed using GraphPad Prism 10. The results are presented as the fold increase of the mean and standard error of the mean for 6 replicates and were normalized to the normal control in the respective group (fold increase = treatment group/normal control).

In bone formation markers, the AA group exhibited a 3-fold increase in OCN [0.36(0.15) ng/mL] and a 5-fold increase in OPN [1.38(0.75) pg/mL] compared to the NC [OCN: 0.13(0.03) ng/mL, OPN: 0.52(0.14) pg/mL], and these values were higher than those in the CA group [OCN: 0.22(0.09) ng/mL, OPN: 0.96(0.50) pg/mL]. Despite no statistical significance, OCN levels were slightly higher in AW [0.24(0.11) ng/mL] compared to CA, while OPN levels in AW [0.60(0.23) pg/mL] were lower than those in CA, as shown in Table 3 and Figure 2. For bone resorption markers, RANKL levels were significantly higher in the AA (*p* < 0.01) and CA (*p* < 0.05) groups, [21.62(3.65)] and [19.1(3.77)], respectively, compared to NC [6.80(1.10)]. TRAP levels, however, were significantly lower (*p* < 0.05) in the CA group [0.11(0.02) pg/mL] compared to NC [0.25(0.03)]. FGF23 levels were significantly higher in the AW group [14.06(1.60) pg/mL] compared to CA (*p* < 0.01) and AA (*p* < 0.05). Antioxidant GPx levels were significantly higher in the AW group [21.04(1.93) pg/mL] compared to NC (*p* < 0.01), AA (*p* < 0.001), and CA (*p* < 0.0001), as demonstrated in Table 3 and Figure 2.

## 4. Discussion

Alcohol use represents a substantial global public health concern, contributing to a spectrum of issues including dependence and withdrawal. It is associated with a plethora of complications, including bone-related problems [17,36]. Chronic alcohol abuse can lead to dependency, characterized by tolerance and withdrawal [37]. While some studies suggest that moderate alcohol consumption may benefit cardiovascular health [38,39] and bone [33], chronic intake of alcohol might be damaging [40]. Given the global increase in lifespan [41], addressing bone health is crucial, as complications like fractures [42] can be fatal.

The ethanol model employed in our study has been validated to simulate alcohol dependence and withdrawal effects [43]. While we previously referenced moderate drinking as potentially beneficial to bone health, our study does not aim to affirm or dispute this; rather, our focus is on exploring the impact of alcohol administration and withdrawal on bone biochemistry. For acute intraperitoneal injections, we administered a dose of 2.5 g/kg in rats, which translates to an approximate human equivalent dose of 418.93 mg/kg. The decision to cull the animals 6 h after the last ethanol administration was based on a previous validation study by Kumar et al. [35,43], which demonstrated that this timeframe effectively induces withdrawal symptoms and allows for the study of early withdrawal effects in experimental models.

Micro-CT has been widely used to study the quantitative changes in bone quality and structural characteristics [44]. Micro-CT assessment is reliable for analyzing rat trabecular structure as it is thinner than human bone [45]. In our study, no significant differences were observed in trabecular bone parameters among the groups. This finding is in accordance with a previous study which also observed no significant difference in bone trabecular microstructure in mice treated with 15% ethanol for 14 days [46]. Contrary to our expectations [47], the acute alcohol (AA) group showed slightly higher connectivity density (Conn.D) compared to the normal control (NC), chronic alcohol (CA), and alcohol withdrawal (AW) groups. This finding may suggest a transient effect of acute alcohol exposure on trabecular connectivity. However, the AA group exhibited the lowest bone volume to total volume ratio (BV/TV) and trabecular thickness (Tb.Th) across all experimental and control groups. These results are consistent with a previous study indicating that alcohol exposure can lead to decreased bone volume and trabecular thickness [48] which may have implications for bone strength and fracture risk. A study on rats fed with three different alcohol doses ad libitum, 25%, 30%, and 35%, also showed significant thinning of trabecular bone after 17 weeks of treatment [49]. There were no changes observed in trabecular number, structural model index, or trabecular separation in the treated groups, suggesting that the effects of acute alcohol exposure on these parameters may be more variable or subtle. Also, trabecular bone appears to be less sensitive to alcohol dosage effects compared to cortical bone in rats, as shown in a previous study [49]. In addition, it is worth mentioning that our study employed intraperitoneal injection and dosages consistent with rat models of AUD [50]. However, despite utilizing a relevant model, the duration of exposure and dosage might not have been optimal for detecting significant changes in trabecular morphology. Future studies could explore longer exposure durations and higher dosages to better simulate chronic alcohol consumption and its effects on bone health.

Interestingly, the AA group exhibited a significant increase in cortical thickness compared to the other groups, which contradicts a previous report showing lower cortical thickness [49]. This finding may be attributed to compensatory mechanisms triggered by acute alcohol exposure or alterations in bone remodeling dynamics. Otherwise, the AA group showed significantly lower total cross-sectional area (Tt.Ar) and cortical area (Ct.Ar) compared to the CA group, which could indicate a reduction in overall cortical bone size in the AA group. Furthermore, the cortical area fraction (Ct.Ar/Tt.Ar) was significantly lower in the AA group compared to the NC, CA, and AW groups, suggesting a decrease in the proportion of cortical bone relative to total bone area. These findings are consistent with previous observations that showed a reduction in cortical thickness after chronic ethanol exposure for 17 weeks [16,49]. This cortical thinning is possibly due to increased resorption at the endocortical surface or decreased formation at the endosteal or periosteal surface of the femur [51], with supporting evidence from histomorphometric changes (reduced cortical bone area, bone formation rates, and mineral apposition rates) in an alcohol-fed group from a previous study [52]. Cortical bone thickness and cortical area are typically proportional; however, we observed that the AA group exhibited a higher cortical thickness (Ct.Th) but a lower cortical area (Ct.Ar). Exceptions to this relationship can occur due to factors such as variations in periosteal apposition rates, endosteal resorption rates, and alterations in bone geometry, which can contribute to discrepancies in measurements of cortical thickness and cortical area [53].

Many studies have reported a dramatic adverse effect of alcohol on bone mechanical properties [54,55]. However, in our study, we did not observe any significant difference in the alcohol-fed or withdrawal group compared to the control, which is in agreement with an earlier study [56]. Possible reasons for this could include the dosage or duration of alcohol exposure or the specific characteristics of our experimental model as mentioned in the previous section.

Osteocalcin (OCN) and osteopontin (OPN) are crucial proteins synthesized by osteoblasts and play pivotal roles in bone mineralization. In this study, we observed no significant changes in bone formation parameters, OCN and OPN, in all groups. However, we noted higher levels of both OCN and OPN in the AA group compared to the NC group. Even though it was not aligned with previous work [57,58], this finding could potentially suggest that acute alcohol exposure can transiently elevate bone turnover markers. RANKL plays a crucial role in bone remodeling by promoting the differentiation and activation of osteoclasts, leading to bone resorption. Our study observed a significantly higher level of receptor activator of nuclear factor kappa-Β ligand (RANKL) in both the AA and CA groups compared to the NC group, which could indicate a potential stimulatory effect of alcohol on osteoclastogenesis, consistent with previous findings [59,60]. TRAP is an enzyme secreted by osteoclasts. Conversely, we observed lower levels of tartrate-resistant acid phosphatase (TRAP) in the CA group compared to NC. The decreased TRAP levels in the CA group may indicate a compensatory response to decreased bone resorption in chronic alcohol exposure, aimed at preserving bone integrity.

Fibroblast growth factor 23 (FGF23) plays a critical role in regulating phosphate homeostasis and vitamin D metabolism, promoting phosphate excretion, and inhibiting the production of active vitamin D to maintain serum phosphate levels. Dysregulation of FGF23 has been implicated in various bone disorders, including osteoporosis. Studies have shown that higher levels of FGF23 are associated with reduced bone density [61,62]. Elevated FGF23 levels have also been observed in alcoholics [63]. However, our study did not observe any changes in bone FGF23 levels in the group treated with acute and chronic alcohol. This finding corresponds with previous research, which reported elevated levels of FGF23 mRNA expression in the liver but did not find similar changes in other organs, including bone [30]. Notably, we revealed a significant elevation of FGF23 levels in the AW group compared to both the AA and CA groups. This suggests a unique response to alcohol cessation, possibly due to disruptions in mineral metabolism and phosphate homeostasis during withdrawal. The observed increase in FGF23 levels during withdrawal could also reflect bone-specific adaptations to restore mineral homeostasis or respond to changes in systemic factors like hormonal shifts or metabolic adjustments. However, further investigation is necessary to comprehensively explore these mechanisms, as they lie beyond the current scope of our study.

Glutathione peroxidase (GPx), an antioxidant enzyme, is crucial for protecting cells from oxidative damage by catalyzing the reduction of hydrogen peroxide and organic hydroperoxides. It scavenges reactive oxygen species (ROS) to prevent cellular damage, which can contribute to the pathogenesis of bone disorders. In this study, we observed increased levels of GPx in the AW group. This may indicate an adaptive antioxidant response to mitigate alcohol-induced oxidative damage during the withdrawal period.

The clinical implications of this study highlight the importance of proactive patient management and rehabilitation for individuals with a history of alcohol consumption. Regular bone health assessments, including monitoring antioxidants such as glutathione peroxidase, FGF23 levels, and bone remodeling markers like RANKL, could be beneficial for early detection and intervention in alcohol-induced bone loss. Tailored treatments, such as fracture risk assessments and targeted rehabilitation programs, are essential to enhance bone strength and reduce fracture risk in these patients. Education on alcohol cessation and lifestyle modifications, alongside long-term follow-up, are pivotal in preserving skeletal integrity and overall health in this vulnerable population.

Our study has several limitations. Firstly, it lacks bone mineral density (BMD) and bone mineral content (BMC) measurements, which are crucial for predicting bone strength and diagnosing osteoporosis. Additionally, histomorphometric studies, essential for explaining certain mechanisms, were not conducted. However, our study is the first, to our knowledge, to utilize this alcohol model of alcohol dependence and withdrawal to explore the relationship between alcohol consumption and bone health. It provides supporting evidence for the widely accepted notion that long-term alcohol consumption negatively impacts bone health. Future research should aim to elucidate the pathogenesis of alcohol-induced osteoporosis by incorporating additional parameters such as bone densitometry (DXA) assessments, serum mineral levels (calcium, phosphate), hormonal levels (parathyroid hormone and steroids), oxidative status parameters, and histomorphometric studies.

## 5. Conclusions

**Figure 3:**
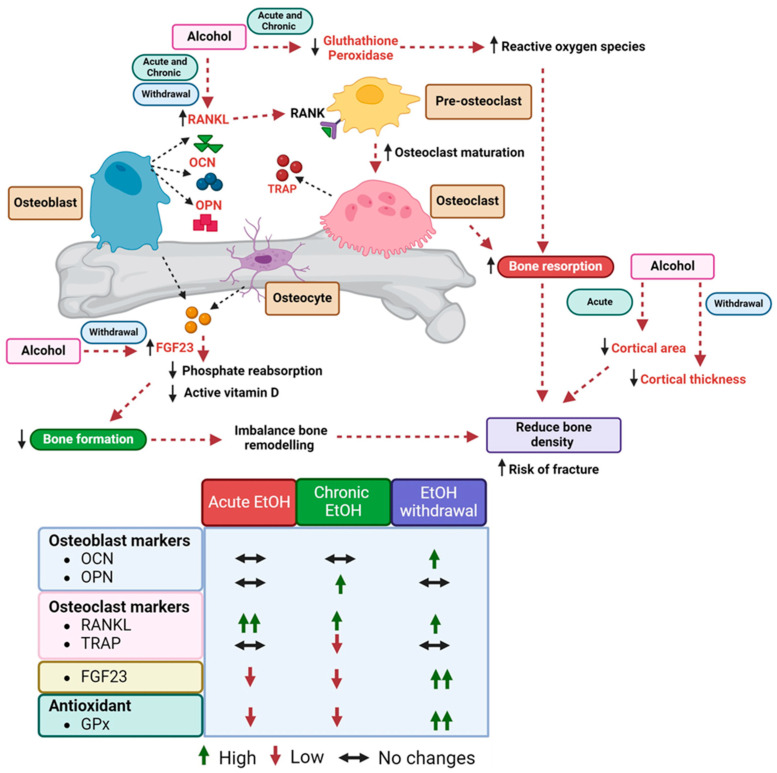
Summary of the bone remodeling protein, FGF23, and antioxidant relationship among alcohol-treated groups and normal control. Abbreviations: EtOH: alcohol, OCN: osteocalcin, OPN: osteopontin, RANKL: receptor activator of nuclear factor kappa-Β ligand, TRAP: tartrate-resistant acid phosphatase, FGF23: fibroblast growth factor 23, GPx: glutathione peroxidase.

In conclusion, our study provides valuable insights into the impact of alcohol consumption on bone health (Figure 3). Acute alcohol exposure transiently increases cortical thickness but reduces cortical area, while withdrawal is associated with decreased cortical thickness. Concurrently, increased RANKL levels observed across all groups suggest enhanced osteoclast activity, potentially exacerbating bone resorption. Elevated FGF23 levels during alcohol withdrawal and reduced antioxidants, such as GPx, during acute and chronic alcohol consumption indicate multifaceted mechanisms underlying alcohol-induced bone damage. Our findings highlight the need for further research to understand the mechanisms underlying alcohol-induced bone damage and to explore potential interventions to mitigate its effects on bone health.

## Figures and Tables

**Table 1:** The effects of alcohol administration and withdrawal on the trabecular bone of the left femur.

Mean (SEM)	Treatment Groups			
	NC	AA	CA	AW
Tb.N (1/mm)	1.40 (0.13)	1.40 (0.15)	1.51 (0.11)	1.52 (0.18)
Conn.D (1/mm^3^)	632.8 (63.01)	703.71 (75.17)	678.23 (77.24)	690.34 (95.94)
SMI	2.03 (0.05)	2.14 (0.08)	2.04 (0.04)	2.19 (0.10)
BV/TV (%)	5.85 (0.55)	5.51 (0.69)	6.57 (0.72)	6.79 (1.07)
Tb.Th (mm)	0.042 (0.001)	0.039 (0.001)	0.043 (0.004)	0.044 (0.002)
Tb.Sp (mm)	0.59 (0.02)	0.56 (0.04)	0.59 (0.02)	0.56 (0.03)

Abbreviations: NC: normal control group, AA: acute alcohol group, CA: chronic alcohol group, AW, alcohol withdrawal group. Tb.N: trabecular number, Conn.D: connectivity density, SMI: structure model index, BV/TV: bone volume to total volume ratio, Tb.Th: trabecular thickness, Tb.Sp: trabecular separation. The results are presented as mean and standard error of mean for 6 replicates.

**Table 2:** The effects of alcohol administration and withdrawal on the biomechanical strength of the left femur.

Mean (SEM)	Treatment Groups			
	NC	AA	CA	AW
Load (N)	104.13 (4.53)	122.31 (8.73)	105.41(7.36)	98.54 (1.24)
Stress (N/mm^2^)	596.50 (21.53)	742.01(55.03)	655.94 (38.32)	617.92 (10.97)
Displacement (mm)	6.27 (0.08)	6.53 (0.13)	6.59 (0.09)	6.47 (0.08)
Strain (%)	6.19 (0.08)	6.99 (0.28)	6.93 (0.27)	6.55 (0.19)
Stiffness (N/mm)	94.84 (4.82)	118.77 (14.44)	126.94 (13.90)	100.38 (4.99)
Young’s modulus (N/mm^2^)	58,819.35 (3078.85)	69,215.97 (8791.08)	71,770.88 (7520.34)	59,703.37 (2899.15)

Abbreviations: NC: normal control group; AA: acute alcohol group; CA: chronic alcohol group; AW: alcohol withdrawal group. The results are presented as mean and standard error of mean for 6 replicates.

**Table 3:** The effects of alcohol administration and withdrawal on bone remodeling markers, an antioxidant, and FGF23.

Mean (SEM)	Groups			
	NC	AA	CA	AW
OCN (ng/mL)	0.13 (0.03)	0.36 (0.15)	0.22 (0.09)	0.24 (0.11)
OPN (pg/mL)	0.52 (0.14)	1.38 (0.75)	0.96 (0.50)	0.60 (0.23)
RANKL (pg/mL)	6.80 (1.10) ^a,b^	21.62 (3.65)	19.1 (3.77)	15.17 (2.39)
TRAP (pg/mL)	0.25 (0.03) ^b^	0.23 (0.05)	0.11 (0.02)	0.17 (0.04)
FGF23 (pg/mL)	9.17 (0.44)	6.76 (1.80)	5.66 (1.40)	14.06 (1.60) ^a,b^
GPx (pg/mL)	11.97 (1.42)	10.88 (0.73)	5.66 (0.70)	21.04 (1.93) ^a,b,c^

Abbreviations: NC: normal control; AA: acute alcohol; CA: chronic alcohol; AW: alcohol withdrawal. OCN: osteocalcin, OPN: osteopontin, RANKL: receptor activator of nuclear factor kappa-Β ligand, TRAP: tartrate-resistant acid phosphatase, FGF23: fibroblast growth factor 23, GPx: glutathione peroxidase. ^a^ significant difference (*p* < 0.05) compared to AA, ^b^ significant difference (*p* < 0.05) compared to CA, ^c^ significant difference (*p* < 0.05) compared to NC. The statistical test was performed using GraphPad Prism 10. The results are presented as mean and standard error of mean for 6 replicates.

## Data Availability

All data are presented within this manuscript.
